# Judgments of mathematical beauty are resistant to revision through external opinion

**DOI:** 10.1002/pchj.556

**Published:** 2022-05-01

**Authors:** Haoxuan Zhang, Semir Zeki

**Affiliations:** ^1^ Laboratory of Neurobiology, Division of Cell & Developmental Biology University College London London UK

**Keywords:** aesthetic judgments, biological beauty, neuroaesthetics, mathematical beauty

## Abstract

We here address the question of the extent to which judgments of mathematical beauty (which we categorize as biological beauty) are resistant to revision through external opinion. A total of 100 mathematicians of different national and ethnic origins were asked to rate 60 mathematical equations for their beauty; after being presented a fictitious “expert rating,” they were asked to re‐rate the same equations. Results showed that the judgments of mathematical beauty had a high level of resistance to external opinion. This is in line with the resistance to revision of a judgments for other categories of biological beauty.

## INTRODUCTION

Mathematicians commonly exalt the beauty of mathematical formulae; some, such as Bertrand Russell (1919), have declared that mathematics possesses “not only truth but supreme beauty” whereas others such as Herman Weyl, if pressed to choose between truth and beauty in a mathematical formula, have declared a preference for beauty. The physicist Paul Dirac (1940) developed a theory of mathematical beauty and proposed that it was the beauty rather than the simplicity of a mathematical formula that was a guide to its veracity. But what does the beauty of a mathematical formula consist of? Immanuel Kant supposed that a mathematical formula is experienced as beautiful because “it makes sense” (Breitenbach, 2013), which of course begs the question of what it makes sense to. One possibility is that it makes sense because it obeys the rules of the brain's logical systems (Zeki et al., 2014); because one assumes that these are the same for all brains, regardless of ethnic and cultural groupings, it is reasonable to assign mathematical beauty to the category of biological beauty, just like the beauty of human faces or bodies, as opposed to artifactual beauty (Zeki et al., 2018; Zeki et al., 2014). This may seem strange; unlike the experience of beauty in human faces or figures, mathematical beauty is perhaps among the most extreme examples of the experience of beauty that is dependent upon culture and learning; no one who is not conversant with the language of mathematics is likely to be able to judge mathematical formulae aesthetically. One test of this would lie in psychophysical experiments to determine the extent to which aesthetic judgments in mathematics are hostage to peer opinion. In general, a characteristic of biological beauty is that, unlike artifactual beauty, it is more resistant to change in light of external opinion; hence, the Bayesian system of belief‐updating should be relatively less effective for mathematical beauty than for aesthetic experiences that belong in the artifactual category (Zeki & Chén, 2020). We have tested this hypothesis here, in light of recent psychophysical studies which have demonstrated that there is indeed greater resistance to modifying aesthetic judgments of stimuli belonging to the biological category compared to ones belonging to the artifactual one (Bignardi et al., 2020; Glennon & Zeki, 2021; Vessel et al., 2018). To undertake our study, we asked mathematicians to rate the beauty of 60 mathematical equations collected for a previous study (Zeki et al., 2014) and studied the extent to which they changed their rating in light of external “peer” opinion.

We have used the same approach here as we did for studying the appreciation of the beauty of human faces and of abstract paintings, where we found that a revision of opinion about the aesthetic status of a face was about twice as unlikely as a revision of opinion about the aesthetic status of abstract paintings, which fall firmly in the artifactual category (Bignardi et al., 2020).

## MATERIALS AND METHODS

### Participants

One hundred participants (60 female) were recruited from mathematical departments of universities worldwide or directly from our knowledge of them; their ages ranged from 20 to 51 (*M* = 23.0, *SD* = 5.68), and all had majored in mathematics, applied mathematics, or statistics. The majority were nationals of the UK (*n* = 40) and various European countries (*n* = 38) whereas the rest were from Asia (*n* = 13), the Middle East (*n* = 5), and South America (*n* = 4). All participants gave their consent after being informed of the objectives of the study, which had been approved by the Ethics Committee of University College London.

### Stimuli

We used the 60 mathematical formulae that were used in a study of the neural correlates of the experience of mathematical beauty (Zeki et al., 2014). As in that previous study, the equations were displayed on a screen in white font on a dark gray background (CMYK 0.00, 0.00, 0.00, 0.80). The base size of the image was 600 × 200 pixels; the width and height of each image were adjusted to fit the different sizes of the equations to produce more visually uniform stimuli.

### Procedure

The experimental procedure was designed using PsychoPy 3.0 software (Peirce et al., 2019), and the experiment was conducted through the Pavlovia.org online research platform. Before starting the experiment, demographic information was collected from the participants regarding their gender, age, and nationalities. Subjects first viewed a sample equation and were asked to complete a sample trial; they were told that the objective of the experiment was to assess the degree to which they would revise their rating of the beauty of the formulae in light of feedback from external experts about the aesthetic status of these formulae. They were not, however, told that the expert ratings were fictitious.

The experiment consisted of 60 trials, each with one mathematical formula (the formulae are available in the supplementary details of the article by Zeki et al., 2014.) These were displayed in random order to prevent order effects. Each trial had two components: (1) a rating of how beautiful the equations were judged by the subject and (2) a rating for how well the equations were understood. Both ratings were given separately, using a scale of 0 (*not beautiful at all*) or (*not understood at all*) to 10 (*very beautiful*) or (*very well understood*). In Part 1 of the experiment, the beauty and understanding ratings were collected via the slider provided in PsychoPy 3.0; to prevent any misclick, the slider was activated 5 s after the equations appeared on the screen, where they remained until ratings were given, which ensured that sufficient time was provided for participants to study the equations and respond. In Part 2, participants were given a fictitious “expert beauty rating” and were told that it was the average beauty rating given to that equation by mathematical experts; in reality, the ratings had been computer‐generated. The original rating given by the participants was not displayed to them when they were asked to re‐rate the equation in light of external opinion. Following previous work of Zaki et al. (2011), Bignardi et al. (2020), and Glennon and Zeki (2021), the fictitious “expert” ratings were derived from the beauty ratings given by the actual participants (discussed below).

We used fictitious rather than real ratings because we consider mathematical beauty to fall under the category of biological beauty (see Zeki & Chén, 2020; Zeki et al., 2014). We therefore expected ratings to be consistent across individuals; hence, the use of real ratings would have been less effective in swaying opinion of the aesthetic status of the equations. When the participants’ first beauty rating was less than 4 (*not beautiful*), the displayed fictitious “feedback” rating had a 50% chance of being the same (*no change*) and a 25% chance of being 2 or 3 points higher. Similarly, where participants had rated the equation higher than 6, the “fictitious” rating had a 50% chance of being the same and a 25% chance of being 2 or 3 points lower. Finally, when the initial rating given was between 4 and 6, the fictitious rating had a 50% chance of being 2 or 3 points higher and 2 or 3 lower (with a 12.5% chance for each) whereas there was a 50% chance of it being the same as the initial rating given by the subject. Table 1 shows how the fictitious ratings were generated using the aforementioned probabilities. In summary, there was on any given occasion always a 50% chance for the fictitious rating to be the same as the initial beauty rating given by the participant. The rating was rounded up to the nearest integer before being displayed to the participants.

**TABLE 1:** The probability distribution of fictitious ratings, Δ*E*

Input	First beauty rating										
	First beauty rating < 4			4 < First beauty rating < 6					First beauty rating > 6		
**Possible Δ*E***	0	2	3	0	−2	−3	2	3	0	−2	−3
**Probability of the Δ*E***	50%	25%	25%	50%	12.5%	12.5%	12.5%	12.5%	50%	25%	25%
**Output**	Fictitious rating = first beauty rating + Δ*E*										

Participants were asked to rate the same equation again immediately after seeing the fictitious “peer” ratings. They were free to either maintain their original rating or give a new one, to both questions.

### Analysis

To ascertain whether external feedback opinion influences the beauty ratings that subjects gave to the equations, responses were divided into two groups: in one group (Group A) were responses in which the feedback rating was identical to the rating given by the subjects; Group A served as a control for the second group (Group B), which was divided into two subgroups according to whether the feedback rating was above or below the initial rating given by the subjects. There were 3,016 ratings in Group A and 2,984 ratings in Group B. To test whether the rating changes (RCs) were derived from normally distributed populations, we submitted the data from the three groups (Group A and the two subgroups of B) to the Lilliefors test to verify whether the RC (second beauty rating−first beauty rating) came from normally distributed populations. The possible effect of gender on the rating was also inspected via a Kruskal–Wallis one‐way analysis of variance.

To investigate the magnitude of RC induced by external feedback opinion, two variables were defined: the RC itself and the difference between the first beauty rating and the external feedback rating (Δ*E*). To facilitate the analysis and grouping, we introduce the term “|RC|” which defines the absolute magnitude of RC; values of RC and Δ*E* were calculated according to the following formula:

RC=second beauty rating−first beauty rating

ΔE=ficticious"expert"rating−first beauty rating

Our main prediction was that external information will not influence aesthetic judgments of mathematical beauty because we hypothesized that mathematical beauty belongs to the biological category (see Zeki et al., 2014). This was tested by the Kruskal–Wallis analysis; an interval plot and multiple comparison tests were applied to both the RC and to within‐subject analyses to determine whether external information affects the magnitude and direction of RC. The RCs in the three groups were compared to check if the fictitious feedback altered the distribution and the mean ranks of the RC. Next, a Spearman rank correlation coefficient was calculated between the two variables: RC (which refers to how much subjects changed their rating) and Δ*E* (which refers to how much we tried, through the fictitious rating, to manipulate their judgment). This allowed us to learn to what extent RC and the feedback are monotonically related and establish the difference between the fictitious “expert rating” and the initial beauty rating (Δ*E*). The result, the correlation coefficient [*r*_s_], reflected how well the two variables were monotonically correlated (i.e., how the magnitude of the rating change correlated with the magnitude of the external fictitious information).

In addition to the main analysis, a regression analysis was undertaken to check if the levels of understanding of the mathematical equations influence the magnitude of RC; that is, whether better understanding of an equation improves the robustness of the initial aesthetic judgment of mathematical formulae judgments, thus making it more resistant to outside influence.

We used the mean minus one (MM1) analysis (Vessel et al., 2018) to investigate the average agreement in mathematical beauty rating among subjects. To calculate the MM1, the correlation between two variables—(1) the beauty rating given by one subject for each equation and (2) the average beauty rating given by all other subjects for the same equation—was computed. This was repeated for all subjects and all equations and produced an individual preference score for each subject; the latter reflects whether an individual subject's beauty ratings for all the equations were in agreement with the mean rating derived from all other subjects for the same equations (Germine et al., 2015; Sutherland et al., 2020). The across‐observer average MM1 score was acquired by applying Fisher *r* to *z* transformation to the individual preference score (*r* value), calculating the mean and transforming back to the *r* value again. Compared to just taking the mean of individual *r* values, the transformations result in less biased estimates of between‐subject average agreement (Bronstad & Russell, 2007), with a higher MM1 value suggesting better agreement between beauty ratings of mathematical equations.

The more widely used interclass correlation coefficient (ICC) (Bi & Kuesten, 2012) was used as an additional check on the results derived from the MM1 analysis. ICC indicates the level of agreement in aesthetic rating given to the formulae by different subjects. In this analysis, the degree of the absolute agreement in beauty ratings, expressed as the average of 100 independent ratings per equation made by 100 participants, was evaluated. A higher ICC value represents a higher uniformity among subjects in beauty ratings for each mathematical equation and vice versa. All analyses were conducted in MATLAB R2020b and RStudio Desktop 1.4.1106.

## RESULTS

### Mean beauty and understanding ratings

The initial and second beauty ratings had means of 5.73 and 5.68 (*SD*s = 2.72 and 2.69), respectively; this shows that there was a high correlation between the second and first beauty ratings, *r*s(5,998) = .91, *p* < .001. The mean understanding rating for the mathematical equations was 5.86 (*SD* = 3.34), suggesting that equations were well distributed according to the ability to understand. As in our previous study (Zeki et al., 2014), a significant positive correlation between understanding level and beauty rating was observed, *r*(5,998) = .60, *p* < .001. The average absolute difference between the first and second beauty ratings was 0.40 (*SD* = 0.74), suggesting a low level of RC across all trials. The feedback Group B (*n* = 2,984) had a significantly higher mean RC (0.56), *p* < .001, compared to the RC of the control Group A (0.28) (*n* = 3,016), for which the external feedback was identical to their original rating. The *SD* of RC in the feedback Group B (0.80) was also numerically higher than that in Group A (0.66). These results show that external feedback resulted in RCs in 21% of the examples in that they revised their judgment of the beauty of the equations. In these 21% of cases, external feedback led to an increase in the magnitude of the RC. In the remaining 79%, the RC was less than 0.77; we regard this as insignificant because it can be the result of errors from inaccurate clicks or rounding‐up processes during fictitious rating generation. The criterion of 0.77 was adopted from the 90% quartile of RC produced by Group A, which reflects 90% of error generated during the process.

The Kruskal–Wallis test on gender led to the rejection of the null hypothesis that the beauty rating given by the two genders came from the same population distribution, H(1) = 9.552, *p* = .002. In other words, gender had an effect on the first beauty rating, which was also reflected by the mean beauty ratings as female participants tended to give lower beauty ratings (5.62 vs 5.80). Next, the same analysis was applied to the changes in beauty ratings made by the two genders to check if the magnitude of RC was influenced by gender, with the result that it was not, H(1) = 49.8, *p* < .001. It appears that female participants tended to have larger RCs compared to male participants, with a mean value of 0.46 and 0.36, respectively.

### Tests for the normality of rating change distributions

The Lilliefors test was applied to the RC in all three groups (Group A and the two subgroups of B) for ratings given positive and negative feedback Δ*E*. The test indicated that none of these groups followed a normal distribution at the 95% CI, *h* = 1, *p* < .001, in that responses were more concentrated centrally; in other words, subjects had been less likely to change their opinion than would have been expected from a normally distributed population. This led us to use a nonparametric analysis for evaluating the effects of external opinion on RC.

### Effect of external opinion on mathematical aesthetic judgments

**FIGURE 1:**
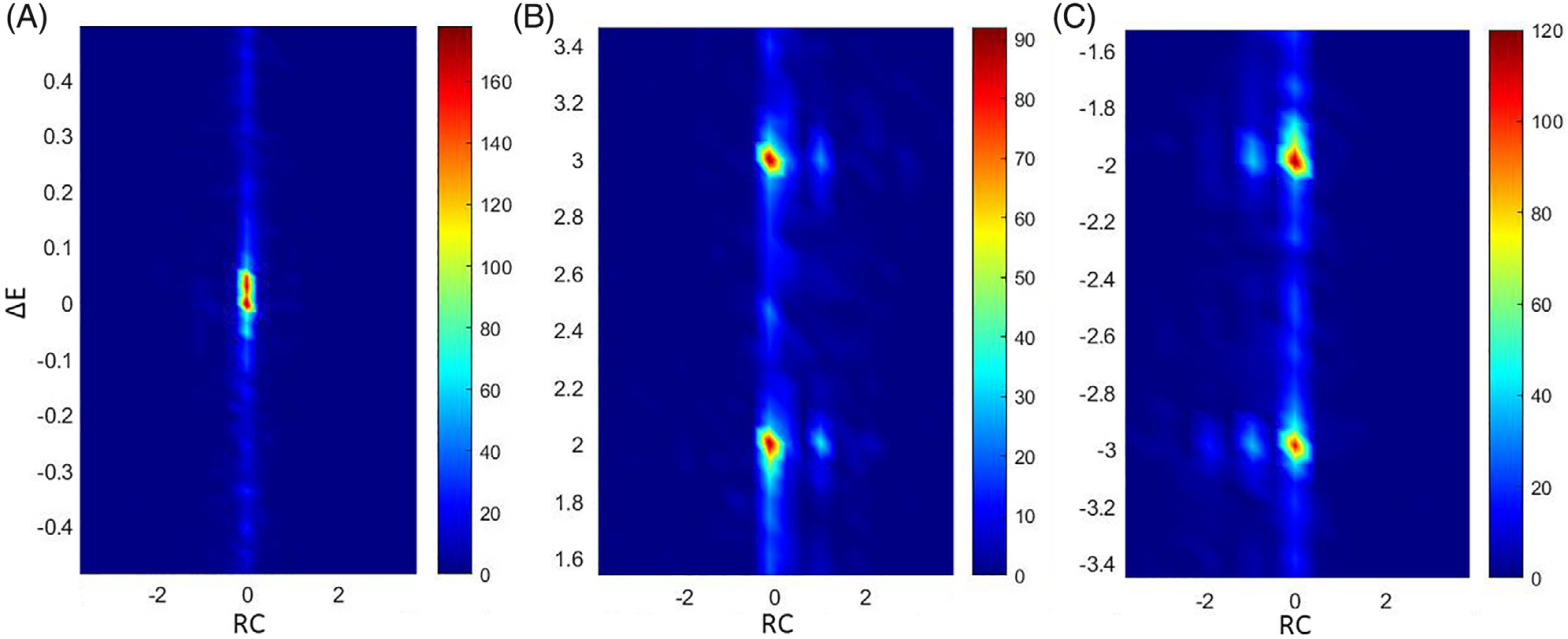
Heat maps of the distribution of rating change (RC) versus Δ*E* in the three groups. Warmer colors represent more RCs at a given value. (A) Distribution in Group A (−0.5 < Δ*E* < 0.5). (B) Distribution in Group B positive Δ*E* subgroup (1.5 < Δ*E* < 3.5) (C) distribution in Group B negative Δ*E* subgroup (−3.5 < Δ*E* < 1.5)

In Figure 1, where the *x*‐axis represents the RC, it is observable that, for all three groups, the great majority of responses had their RCs clustered around zero. This indicates that judgments of mathematical beauty are very resistant to change in light of external opinion; where external opinion had an effect (i.e., where *ΔE* existed) (Figure 1B,C), a portion of the responses was observed to have more positive or negative RCs, as compared to the responses in Group A (Figure 1A).

**FIGURE 2:**
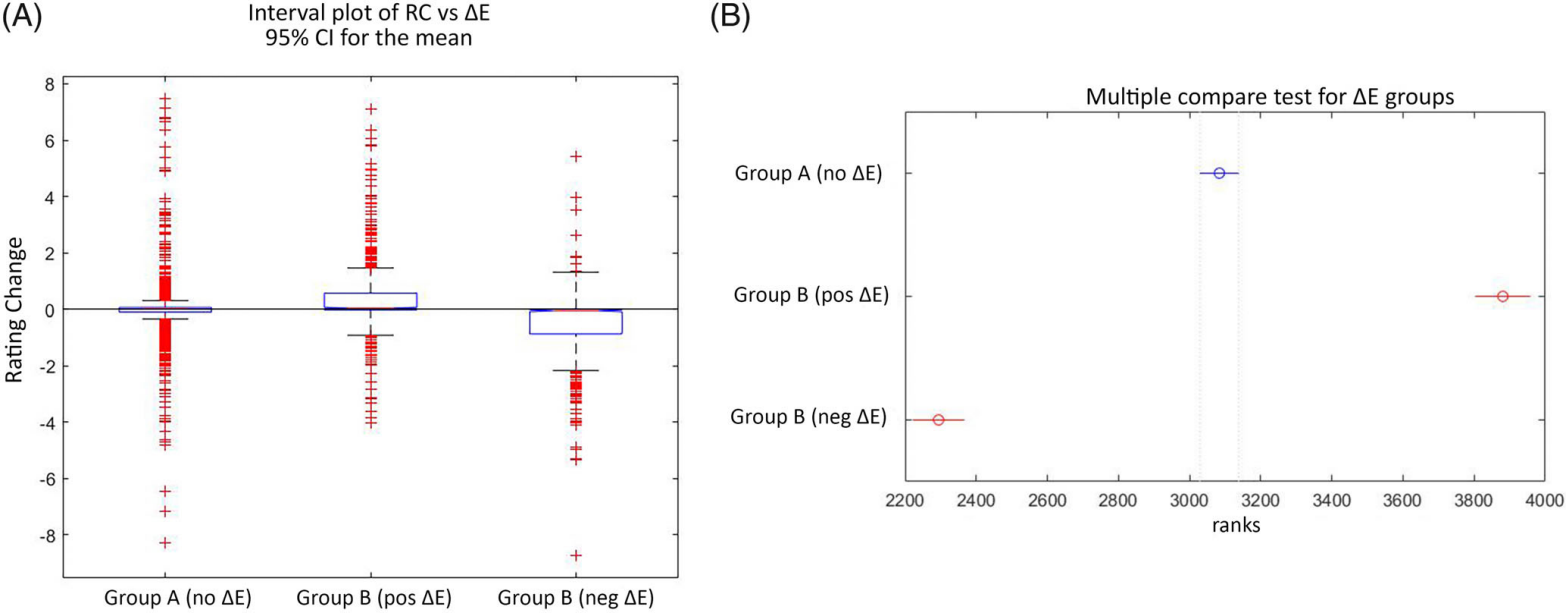
(A) Interval plot of rating change (RC) versus Δ*E* for the three groups. (B) Multiple comparison test results for the three groups; RCs in Group A have mean ranks significantly different from two subgroups of Group B

To evaluate the effect of the external information on the RC, we applied the Kruskal–Wallis test to all three groups. The result showed a significant difference between the means among the three groups, H(2) = 613.02, *p* < .01; in other words, where there was a rating change, this was the result of the presence of Δ*E* and not due to random factors. This conclusion was further supported by the multiple comparison test (see Figure 2B), which shows that both positive and negative Δ*E* feedback groups had mean ranks significantly different from Group A and were also different from each other. The interval plot of Figure 2A shows that in Group A, the upper and lower quartiles were densely packed within a 1‐point range, with mean RCs close to 0. For the positive and negative Δ*E* group, the means were higher and lower than 0, respectively, with significantly wider interquartile ranges. Thus, where Δ*E* was effective in inducing an RC, it affected both the occurrence and magnitude of the RC, though the overall RC was small for both.

**FIGURE 3:**
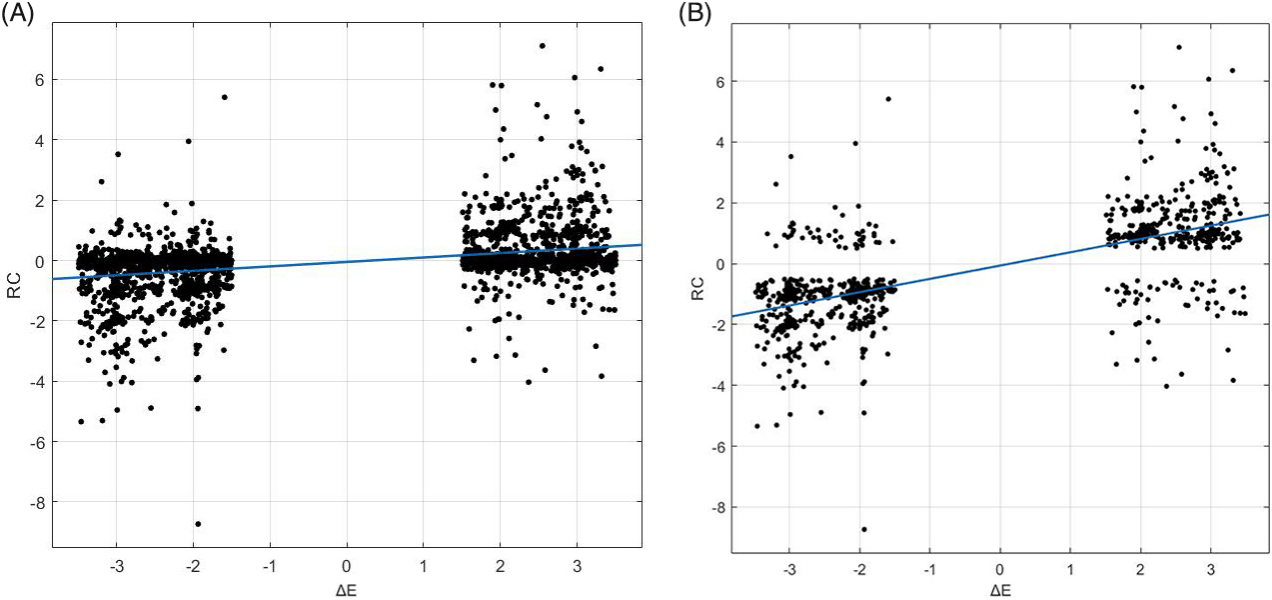
(A) Correlation of rating change (RC) and Δ*E* in the feedback group. (B) Same correlation, with trials without significant |RC| < 0.5 excluded

The Spearman correlation analysis also showed that within the feedback group, RC and Δ*E* had a significant positive correlation, *r*s(2,984) = .391, *p* < .001. In other words, a more positive Δ*E* rendered participants less resistant to external opinion, and vice versa (Figure 3A); considering that there was no significant RC, |RC| < 0.77, for over two thirds of trials (2,068 in 3,047), the exclusion of such trials will render the above correlation numerically higher, *r*s(978) = .672, *p* < .001.

There was a weak negative correlation *r*s between understanding level and RC, *r*s(2,984) = −.112, *p* < .01, suggesting that a better understanding may lead to an even smaller RC.

### MM1

**FIGURE 4:**
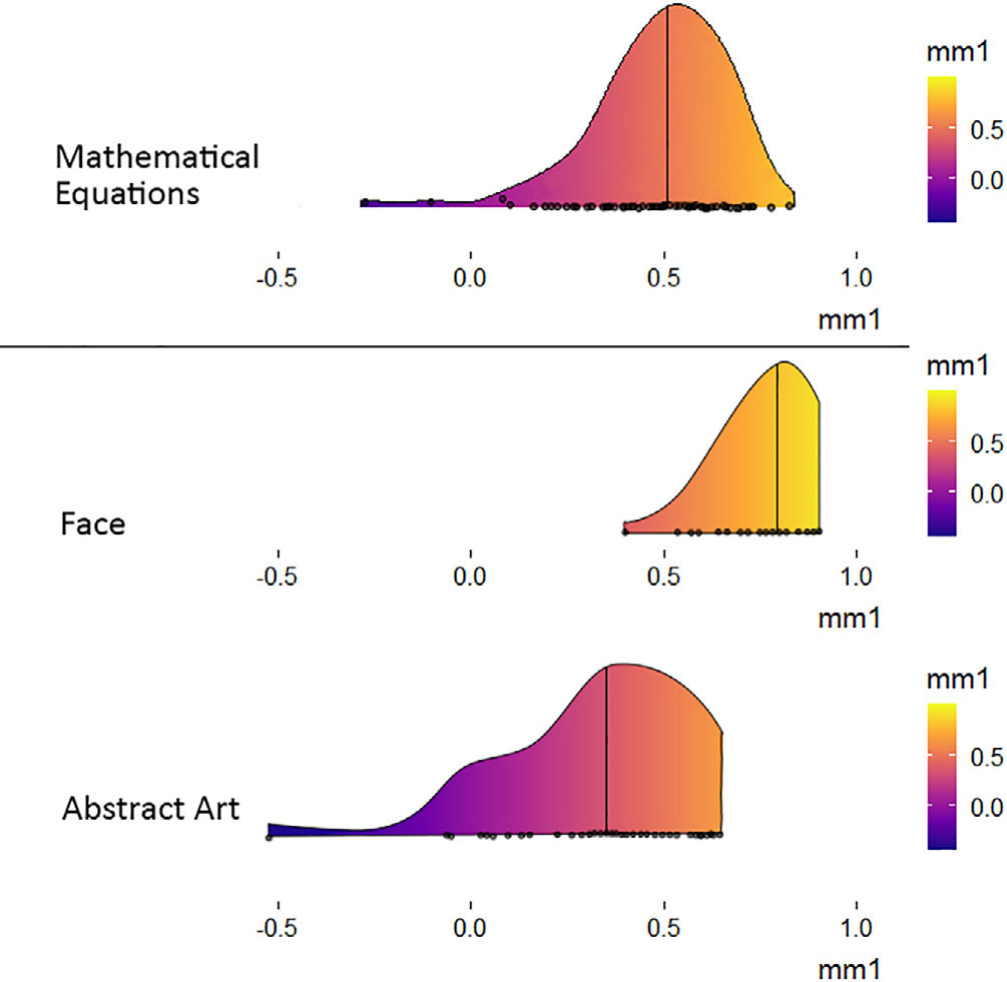
Mean minus one (MM1) plots to show the degree of agreement in beauty ratings across subjects. Data for faces and abstract artworks were adapted from Bignardi et al. (2020). Black dots represent individual preference scores (how well a certain subject's beauty rating agrees with the average rating given by others). Vertical line represents the across‐observer average MM1 score

The MM1 score shows the degree of agreement among subjects in their ratings of stimuli, with an MM1 score above 0.5 indicating good agreement. In this study, the across‐observer average MM1 score showed a good degree of agreement for mathematical beauty, MM1 = 0.509, 95% CI [0.455, 0.528]. This MM1 value is higher than those for abstract works, which were 0.3 in the works by Bignardi et al. (2020) and Vessel et al. (2018); however, it is lower in value than that obtained for other biological categories, which stand at c 0.7 for facial beauty and c 0.6 for landscapes in the study by Vessel et al. (2018) and at 0.81 in the studies by Bignardi et al. (2020) and Glennon and Zeki (2021) (Figure 4).

The extent of agreement between individuals’ aesthetic (or other) judgments is probably a reliable pointer to the extent to which they would revise their judgments in light of external opinion; the higher the agreement, the more resistant they would probably be to revision. The extent of the agreement between individuals’ ratings is better shown by the more widely used ICC, where a value above 0.75 is excellent whereas a value below 0.4 is poor (Cicchetti, 2001). In our study, we obtained a two‐way random, average ICC value of 0.967 at a 95% CI level for beauty ratings of mathematical equations. This shows that there is a very high level of absolute agreement in the aesthetic rating of mathematical equations, thus further supporting the view that mathematical beauty can be categorized as a form of biological beauty. Taken together, these values would appear to justify the correlation between high agreement and high resistance to revision of opinion.

## DISCUSSION

In this study, we extend our previous work on the extent to which opinions formed through different experiences are susceptible to modification or revision through external opinion. At the basis of this work lies our belief that all experiences are interfaced through brain concepts, which we broadly divide into two categories: inherited concepts and postnatally acquired synthetic ones (Zeki, 2009; Zeki & Chén, 2020). In developing this line, we have suggested that in the Bayesian context, inherited biological concepts generate posteriors which are either very, or relatively, more resistant, to modification through further experience or feedback. By contrast, the posteriors generated from acquired concepts are much more hospitable to external influence and opinion (Zeki & Chén, 2020). We furthermore conjectured that within the biological category, the resistance to modification through external opinion is not rigid but graded, with the aesthetic rating for some categories of biological stimuli being more resistant to change through external opinion than others. The studies that we and others have conducted (Bignardi et al., 2020; Glennon & Zeki, 2021; Vessel et al., 2018) support this broad general subdivision. One of the extremes that falls into the biological category is that of color or, more precisely, color categories; it is difficult, if not impossible, to get individuals to change the color category into which they place a surface, even when there are wide changes in the wavelength‐energy composition of the light reflected from them (Zeki et al., 2019). Another, less extreme, category is that of facial beauty, a category for which subjects are about twice as resistant to changing their aesthetic ratings in light of received opinion, compared to artifactual beauty (e.g., abstract paintings) (Bignardi et al., 2020). From the weak correlation between understanding of equations and the rating change, we conclude that the rating change is not necessarily dictated by a lack of understanding.

We have based our experiments on the belief that the aesthetic judgments of mathematical formulations belong in the biological category (Zeki et al., 2014); indeed, the very fact that the veracity of mathematical formulations demands universal assent among mathematicians of all races points to that end. And, though not explicitly enunciated, this is implicit in Plato's belief that mathematical beauty is the highest form of beauty. Paul Dirac's principle of beauty in mathematics is also based on the supposition of a common agreement between mathematicians: “It is the same in all countries and at all periods of time” (Kragh, 1990, p. 288).

It could be argued that because our subjects were professional mathematicians, they may have been in one way or another familiar with these equations, the judgments related to them, or their contexts (as coming, e.g., from famous mathematicians). Although we cannot discount this possibility, we consider it to be unlikely. If mathematicians use (or have used) similar rules to understand and appreciate these formulae, then our point becomes more emphatic because it implies that the rules are general ones, despite the diversity of our mathematicians; this, in turn, would imply that these rules are dictated by brain logic. Moreover, we have shown that understanding had little effect on the RC. Finally, such an interpretation would be contradicted by the fact that in 21% of the cases, the mathematicians did revise their aesthetic judgment in light of external opinion, which suggests that although the resistance to revision of opinion is high, it is not absolute. This leads us to conclude that it is largely the logical system of the brain that is the determining factor in making aesthetic judgments in mathematics, but that there is nevertheless room for doubt in the making of such judgments, because there was an RC in 21% of the examples.

We are truly sorry to have taken the reader on such a complex statistical journey to prove what is essentially a simple point—that the resistance to revision of aesthetic judgment of mathematical formulae in light of external opinion is very high; this in turn justifies our categorization of mathematical beauty in the biological category.

## CONFLICT OF INTEREST

The authors have no competing interests to declare.

## ETHICS STATEMENT

Ethical permission to undertake this study was provided by the UCL Ethics Committee.

## Data Availability

Data available from the authors.
